# A Comparative Review of Typical and Atypical Optic Neuritis: Advancements in Treatments, Diagnostics, and Prognosis

**DOI:** 10.7759/cureus.56094

**Published:** 2024-03-13

**Authors:** Noah J Spillers, Patrick M Luther, Norris C Talbot, Evan J Kidder, Connor A Doyle, Salim C Lutfallah, Alyssa G Derouen, Sridhar Tirumala, Shahab Ahmadzadeh, Sahar Shekoohi, Alan D Kaye, Giustino Varrassi

**Affiliations:** 1 School of Medicine, Louisiana State University Health Sciences Center, Shreveport, USA; 2 School of Medicine, Louisiana State University Health Sciences Center at New Orleans, New Orleans, USA; 3 Department of Anesthesiology, Louisiana State University Health Sciences Center, Shreveport, USA; 4 Department of Pain Medicine, Paolo Procacci Foundation, Rome, ITA

**Keywords:** mog-igg, ivig, plasmapheresis, corticosteroids, aqp4, neuromyelitis optica spectrum disease (nmosd), optic neuritis

## Abstract

Optic neuritis (ON) is a debilitating condition that through various mechanisms, including inflammation or demyelination of the optic nerve, can result in partial or total permanent vision loss if left untreated. Accurate diagnosis and promptly initiated treatment are imperative related to the potential of permanent loss of vision if left untreated, which can lead to a significant reduction in the quality of life in affected patients. ON is subtyped as "typical" or "atypical" based on underlying causative etiology. The etiology of ON can be differentiated when appropriate diagnostic testing is performed. Using history taking, neuroimaging, and visual testing to localize the underlying pathology of ON in a time-sensitive manner is critical in mitigating these unsatisfactory outcomes. Herein, we examine the differences in presentation, pathophysiology, and treatments of typical ON causes, like multiple sclerosis (MS), and atypical causes such as neuromyelitis optica spectrum disorder (NMOSD) and myelin oligodendrocyte glycoprotein (MOG)-immunoglobulin G (IgG) ON. The present investigation places focus on both neuroimaging and visual imaging in the differentiation of ON. Additionally, this review presents physicians with a better understanding of different presentations, treatments, and prognoses of ON.

## Introduction and background

Optic neuritis (ON) is characterized as inflammation of the cranial nerve II [1]. The optic nerve is responsible for the critical conduction of nerve signals from the retina to the lateral geniculate body in the thalamus [2]. ON is often an early manifestation of central nervous system (CNS) inflammation [1]. Inflammation in these critical components of the visual pathway can severely influence patients' vision. Although the initial physical examination of a patient presenting with typical versus atypical ON can seem similar, diagnostic testing in imaging and serological tests can further elucidate the underlying causative pathology [1]. Several etiologies other than multiple sclerosis (MS) are known to cause ON. Two other conditions known to cause ON are neuromyelitis optica spectrum disorder (NMOSD) and myelin oligodendrocyte glycoprotein (MOG)-immunoglobulin G (IgG) ON [1]. These etiologies are grouped into broad "typical" and "atypical" classifications. Typical ON is associated with characteristic retro-orbital pain on extraocular movements and acute monocular vision loss due to an underlying demyelinating lesion, such as those seen in MS [3]. Conversely, ON subtypes are termed atypical ON if the underlying etiology of the condition arises from neuromyelitis optica (NMO), NMOSD, MOG-IgG ON, or other pathologies that are not relevant to the discussion of this review [3]. Atypical ON is generally painless, but patients suffer worse vision prognoses. These atypical etiologies vary in their initial presentation and severity, which can impact long-term outcomes in patient vision. Ophthalmic testing alone is not generally helpful in diagnosing ON [1]. A diagnosis of ON requires several aspects, including careful exam and history taking, visual function testing, optical coherence tomography (OCT), and neuroimaging [1]. Related to advances in immunology and technology in optic nerve imaging, clinicians can rapidly interrogate optic nerve structure and function during these inflammatory events [1]. In this regard, these recent advances enable physicians to identify autoimmune targets for initiating treatment and maximizing patient outcomes [1]. An accurate diagnosis is crucial for preserving vision, preventing neurological dysfunction, and even preventing future organ damage [1]. Quick recognition and diagnosis of ON can lead to prompt treatment, thus aiding in regaining visual function and alleviating symptoms. This review aims to discuss advances in diagnostic imaging tests and explore the different etiologies causing ON and their respective presentations, prognoses, and treatments.

## Review

Presentation

The term ON describes acute unilateral, or less commonly bilateral, inflammation affecting the function of the optic nerve, resulting in a decreased or total visual loss that is generally associated with a painful sensation. Aggravating inflammation can be caused by many factors including trauma, vascular insufficiency, infection, toxins, or autoimmune-mediated demyelination [2]. The population most affected by ON are females aged 20-40 years. Other risk factors for ON are obesity, smoking, MS, the Caucasian race, and living in geographically higher latitudes [4]. A landmark randomized control trial by the Optic Neuritis Study Group (ONSG) in 1992 set the description for typical ON in the literature. This study evaluated 457 patients among 15 clinical centers. It found that 95% of the study group presented with unilateral vision loss and 92% had retro-orbital pain with extraocular movements [5]. Other presenting symptoms of ON include optic disc edema, associated pain, symptom progression over time, preceding vaccination/infection, atrophy of the optic nerve, and history/family history of autoimmune conditions [6].

There are two subtypes of ON: "typical" and "atypical." Typical ON follows the symptoms presented by the ONSG, with unilateral vision loss commonly associated with retro-orbital pain upon extraocular movement. Atypical ON is defined by any aberration of those symptoms, whether by bilateral onset, painless extraocular movements, ON refractory to corticosteroids, or optic nerve head/peripapillary hemorrhages. All of the previously mentioned aberrations are due to underlying conditions such as NMOSD, MOG-IgG ON, or other inflammatory, infectious, or autoimmune disorders, excluding MS [7].

NMO was first discovered by Gault et al. and described as a condition accompanied by severe ON presenting either bilaterally or chronically and often led to blindness [8]. Initial descriptions of NMOSD were believed to be a differential expression of phenotypic features seen in MS. However, the discovery of NMO-IgG aquaporin-4 (AQP4) antibodies ultimately led to the distinction of this pathology from MS [9]. NMOSD affects females over males at a 10:1 ratio. NMOSD is also generally found in non-Whites, and many countries affected by European colonization, such as the West Indies and Australia [9-11]. The median age range of NMOSD is 30-50 years old [12]. NMOSD follows a similar pathology with optic nerve inflammation relapsing and remitting in intervals that are inadequately spaced, resulting in permanent damage or vision loss that is generally worse than that seen in MS-related ON [13]. Characteristic features of ON associated with NMOSD include lesions of the optic chiasm and tracts, bilaterally or rapid succession onset, central or paracentral scotoma, bitemporal hemianopsia, and color blindness [14].

MOG-IgG, similar to NMOSD, was initially believed to be a subtype of MS until a landmark study by O'Connor et al. found that antibodies associated with acute disseminated encephalomyelitis preferentially bound MOG tetramers. Thus, the study concluded that MOG-IgG might be unrelated to MS [15]. Further immunoassay development and studies would delineate MOG antibody disease (MOGAD) from MS and establish it as a separate pathology. Preliminary epidemiological studies of MOG-IgG prevalence suggest that this condition is seen in 1.6-3.4 per 1,000,000 [16], with children accounting for up to half. Males and females appear to be equally affected [16]. Median age onset of the condition is 20-30 years of age [17]. MOG-IgG ON typically presents with severe optic disc edema in approximately 86% of patients and bilateral acute ON with recurrence as high as 50%. In the recurrent cases of this condition, patients can develop chronic relapsing inflammatory optic neuropathy (CRION) [13,17]. Bilateral vision loss could be as high as 50% [18]. These patients tend to have greater vision recovery than those with APQ4-IgG seropositive NMOSD [19].

Diagnostics

Over the years, the development of highly accurate antibody testing, the newfound use of retinal OCT, and the development of time-sensitive treatments for ON have made prompt, accurate diagnosis, a marker of positive patient outcomes, such as a reduction in vision loss [6]. Serological tests are valuable to clinicians when they suspect an autoimmune-mediated subtype of ON. ON can be diagnosed based on clinical exams, paraclinical evidence, and magnetic resonance imaging (MRI). Typical ON presentation may warrant a clinician to consider further diagnostic testing [6]. Likewise, patients who present with clinical signs consistent with atypical ON should also be considered for further diagnostic testing [6].

Conversely, patients who present with hallmark characteristics of ON but do not have appropriate paraclinical testing available or have a retrospective clinical history that illustrates ON, should be considered as "possible ON"[6]. In 2022, a multidisciplinary group of physicians evaluated past diagnostic criteria for ON. The result of this gathering outlined ON in three classifications. Level 1 considers whether the case is likely to be relapsing or just monophasic [6]. Level 2 details ON due to autoimmune conditions with other diseases likely to be monophasic associated with these conditions [6]. Level 3 is dedicated to rare subtypes of ON that are recognized by individual experts but did not reach a consensus [6].

MRI

Diagnosis of ON is typically performed with clinical signs and history. Atypical cases may include a workup of an MRI to assess prognosis on the likelihood of developing conditions such as MS or NMO [20]. MRI is particularly useful due to its ability to show contrast in soft tissues, without radiation exposure, and better delineate the entire visual pathway [21]. The utilization of MRI helps localize demyelinating lesions in various ON. An acute presentation of ON is seen on MRI as a T2-weighted signal change with edema and T1-gadolinium enhancement of the affected optic nerve [22]. Findings include length of the lesion, presence of perineuritis and orbital fat enhancement, and localization and distribution of the lesion(s) within the brain or spinal cord [22]. The MRI findings associated with ON are well defined in a study performed by Onodera et al. as a signal intensity ratio (SIR) average cutoff of 1.119 with sensitivity, specificity, and accuracy of 0.939, 0.840, and 0.870, respectively; and an SIR max level of 1.281 with sensitivity, specificity, and accuracy of 1.00, 0.720, 0.806, respectively [23]. Specifically in typical ON, the lesion length is focal as opposed to longitudinally extensive, which is found in both MOGAD-associated ON and NMOSD-associated ON [22]. Typical ON lesions are commonly orbital and canicular, rather than intracranial [22]. MOGAD-associated ON lesions are typically longitudinal and involve orbital, intracranial, and canicular locations, with the orbital being the most common lesion location [22].

Conversely, NMOSD-associated ON lesions most commonly are located intracranially [22]. In typical ON, optic tract and chiasm involvement is rare [22]. In MOGAD-associated ON, chiasm involvement is usually a result of an extensive optic nerve lesion. However, in NMOSD-associated ON, the chiasm is commonly involved as an isolated lesion [16]. Finally, perineuritis and orbital fat involvement are common in MOGAD-associated ON but rarely seen in typical ON and NMOSD-associated ON [17]. Specific MRI findings help clinicians isolate the specific subtype of ON and provide evidence of the causative etiology, providing further insight as to which serological test should be performed to initiate treatment.

Fundus Exam

The fundus contains the retina, macular, optic disc, fovea, and retinal blood vessels [24]. A fundus exam can be done both by physical exam by an ophthalmologist or through a diagnostic image called fundus photography (FP). In an FP, multiple lenses are used in the indirect ophthalmoscope to capture an image of the inside and backside of the eye, allowing visualization of the fundus [24]. Fundal examination evaluates the presence of edema in the optic disc as a marker of CNS inflammation. Although disc edema suggests inflammation, there are varying amounts of visible edema in different types of ON on clinical exams [22]. In typical ON, there is only infrequent (30%), mild disc edema found, whereas in the atypical ON, MOGAD-associated ON, and NMOSD-associated ON, moderate-severe disc edema is found in 70%-80% of cases, and mild disc edema is found in 36% of cases [22]. In another atypical type of ON, autoimmune glial fibrillary acidic protein (GFAP)-IgG-associated meningoencephalomyelitis, a fundal exam reveals frequent moderate disc edema with the characteristic presence of vitreous and periphlebitis [22].

OCT

OCT is a noninvasive imaging test that uses light waves to take 10-15 micron cross-sections of the retina [25]. These thin cross-sections allow visualization of the individual nerve layers of the retina and optic nerve. OCT can quantify retinal cell loss and axonal damage in acute ON [26]. This imaging modality allows clinicians to initially diagnose diseases of the retina and ON but also allows for the detection of subtle changes to these layers that may not yet be symptomatic to a patient. Specifically, the retinal nerve layers known as the peripapillary retinal nerve fiber layer (pRNFL), macular ganglion cell-inner plexiform layer (mGCIPL), inner nuclear layer (INL), and the outer plexiform layer (OPL) are examined to assess differences in various ON etiologies [22]. pRNFLs are measured at presentation of ON to assess optic disc edema, where it may be found to be of normal width or increased depending on the extent of the edema [22]. Subsequent measures of both pRNFL and mGCIPL show the effects of chronic optic nerve atrophy with the thickening of the INL [22]. In typical ON, all four layers showed evidence of chronic findings. However, in the atypical cause MOGAD-associated ON, the chronic findings of both the pRNFL and mGCIPL were severe, with additional frequent findings of microcystic macular edema [22]. Additionally, in NMOSD-associated ON, similar findings were found compared to MOGAD-associated ON with the addition of severe chronic findings of thinning of the OPL [22].

Visual Evoked Potentials (VEPs)

VEPs are the expression of electrical activity in the visual pathways up the optic nerves and to the calcarine cortex [27]. VEPs are an electrophysiological tool that is extremely useful in following neurological pathologies. These potentials are measured by applying electrodes to the scalp in the occipital region and exposing the patient to a visual stimulus [28]. Measuring these electrical potentials allows physicians to assess the visual pathway noninvasively by detecting pools of neuronal output activity in response to stimuli without the need for the patient to be conscious or attentive [27]. This test is especially clinically useful as a sensitive indicator for visual pathway pathologies due to demyelinating diseases, such as MS or axonal pathologies [27]. Additionally, another interesting use of VEPs is its potential to detect latency delay of potential-peak eye velocity (P-PEV) in the contralateral eye, which can be used to detect subclinical alterations in the impulse conduction along the optic nerve before it presents clinically [29]. In most etiologies of ON, acute findings in P100 latency delay are common [22]. The main differences are seen when examining P100 amplitude loss and extinguished VEP. Acute P100 amplitude loss is common in MOGAD-associated ON but infrequent in typical ON and NMOSD-associated ON [22]. Conversely, acute findings in extinguished VEP are common in NMOSD-associated ON but infrequent in typical ON and MOGAD-associated ON [22].

Pathophysiology and prognosis

Typical ON relates to an idiopathic inflammation of the optic nerve that is often associated with the development of MS. Atypical ON cases arise from afflictions other than MS, including any inflammatory, infectious, or autoimmune disorders [30]. Two atypical ON-associated autoantibody diseases are MOGAD and NMOSD, of which our current knowledge is quickly improving [30]. These diseases can lead to the development of ON or are associated with its occurrence and recurrence.

MS

MS-related ON is a direct result of inflammatory demyelination of the CNS. In 20% of MS patients, ON is the first symptomatic event of demyelination. Approximately 50% of patients with MS will develop ON at some point [31,32]. MS involves focal inflammation that develops into macroscopic plaque(s), leading to damage to the blood-brain barrier (BBB). Furthermore, it creates neurodegeneration with microscopic injury of the CNS involving axons, neurons, and synapses [33]. These two processes largely contribute to the destruction of the CNS macroscopically and microscopically. The plaques found in MS correlate to increased loss of myelin, edema, and axonal injury [33]. On top of the demyelination and inflammation in the brain, the damage to the BBB formulates the autoimmune activation of macrophages and monocytes, thus starting a systemic response in the brain [33]. As a result, microglial cells initiate oxidative bursts destroying myelin in the brain. Furthermore, microglia serve a part in the remyelination of the nerves by clearing myelin debris and enabling oligodendrocyte progenitor cells, which develop into mature myelin-generating oligodendrocytes [34,35]. Studies in the past six years have shown that microglia might play a larger role in MS by not only contributing to the destruction of the CNS but also preventing the remyelination of neurons, thus leading to the worsening of symptoms. More data has also unveiled that B cells might have a larger role in MS patients, as these cells displayed increased release of proinflammatory cytokines compared to healthy controls [36]. While the complexities of MS pathogenesis create difficulties in finding the exact cause, these autoimmune reactions and specifics contribute to the direct development of typical ON.

Typical ON induces pain upon eye movement in 90% of cases but has a relatively good prognosis, in terms of visual acuity [3,37]. Most patients maintain a visual acuity over 20/200 with a progressive visual loss over two weeks before stabilizing. Approximately 72% of patients, based on an ON treatment trial, were able to maintain visual acuity of 20/20 in both eyes at a 15-year follow-up [32].

MOGAD

MOGAD is primarily marked with perivenous and confluent white matter demyelination with an overrepresentation of intracortical demyelinated lesions when compared to MS [38]. This disease holds both antibody-dependent and antibody-independent features: complement deposition, inflammatory T-cell infiltrates, granulocytosis, astrogliosis, microglial activation, moderate axonal loss, and oligodendrocyte precursor preservation [22]. The T cell infiltrate in MOGAD is composed of predominantly CD4+ T cells, as opposed to the MS inflammatory infiltrate, which contains primarily CD8+ T cells [39,40]. In contrast to the complement-initiated damage in NMOSD, MOGAD is presumed to have different initiating factors than complement activation [22,41].

Importantly for a patient presenting with atypical ON, the differentiation between AQP4-IgG and MOG-IgG provides clinically helpful data to determine a diagnosis of NMOSD or MOGAD. This information is crucial in terms of prognosis, as MOGAD-related ON has improved results following treatment compared to NMOSD [42-44]. A study by Chen et al. identified that 80% of MOGAD patients will develop recurrent attacks of ON [45]. At presentation, vision loss can appear profound. However, recovery in MOGAD is more promising than in NMOSD, with patients showing visual acuities with a median of 20/20 and a mean of 20/30 for visual recovery, although it was correlated to steroid treatment [45].

NMOSD

Characterized as an inflammatory CNS syndrome, NMOSD, also known as Devic disease, is associated with the serum AQP4-IgG antibodies and is considered an autoimmune demyelinating astrocytopathy [46,47]. Around 70%-90% of NMOSD patients will be seropositive for AQP4-IgG [22,48]. AQP4 receptors are the most abundant water channel in the brain, spinal cord, and optic nerve, displaying control over water homeostasis [49-51]. They particularly are found most abundantly in astrocytes and ependymal cells lining the ventricles; however, perivascular astrocytes end feet surrounding the CNS vasculature contain the highest numbers of AQP4 [52]. ON occurs in NMOSD through autoimmune attacks from AAP4-IgG, targeting multiple areas, including the optic nerves [53]. Experimental data has suggested that AQP4-IgG is responsible for activating interleukin-6 within epithelial cells, leading to a less effective BBB function [54]. Furthermore, AQP4-IgG binding to the AQP4 receptor leads to endocytosis/degradation and complement activation, rendering the astrocytes nonfunctional [55]. Other cells reliant on astrocytes, such as oligodendrocytes and neurons, begin to lose support, leading to granulocyte infiltration, oligodendrocyte damage, and demyelination [56].

Of the three associated diseases with ON discussed, NMOSD displays the worst prognosis. NMOSD-related ON will lead to worsening results with each recurrent episode [57]. The initial ON attack will leave 20%-30% of patients with 20/200 or worse visual acuity, which is functionally blind, and 70% of patients experiencing relapses will have a visual acuity of 20/200 or worse [58,59]. This prognosis has the most damaging impact on vision, leading to a higher chance of little to no improvement of visual acuity with worsening results after subsequent attacks.

Other Etiologies of ON

Aside from pathologic disease states that are known to cause ON, several drugs and/or risk factors have been associated with the development of ON. ON caused by medication is termed toxic optic neuropathy (TON). The exact pathology of TON is unknown. However, it is generally accepted that most cases of TON are related to some impairment in the tissue’s vascular supply, metabolism, or mitochondrial injury which results in an imbalance of free radicals [60]. In TON, the severity of affliction is usually dose-dependent as well as the duration of treatment with the causative medication [61]. Additionally, cessation of the offending drug typically resolves the ON symptoms. The American Academy of Ophthalmology describes the many drugs that contain ON as a part of their adverse effect profile [60]. Among these drugs, numerous drug classes are shown to have the risk of developing ON. Table 1 highlights the drug classes and nutritional deficiencies shown to have a known risk for developing ON. In addition to TON, nutritional deficiencies can predispose individuals to developing ON. Specifically, deficits in vitamins thiamine (B1), riboflavin (B2), niacin (B3), pyridoxine (B), cobalamin (B12), and folic acid can trigger or augment TON (Table 1) [60].

**Table 1:** Nutritional Deficiencies and Known Drug Classes to Cause Toxic Optic Neuropathy Sources: [60-62]

Nutritional Deficits	Drug Class	Example Drugs
Thiamine (B1)	Alcohols	Methanol, ethylene glycol
Riboflavin (B2)	Antibiotics	Chloramphenicol, sulfonamides
Niacin (B3)	Antimalarials	Hydroxychloroquine
Pyridoxine (B6)	Antitubercular	Isoniazid, ethambutol
Cobalamin (B12)	Antiarrhythmic	Amiodarone
Folic acid	Anticancer	Vincristine, methotrexate
	Antidepressants	Sertraline
	PDE inhibitors	Sildenafil

The prognosis of TON depends on the causative drug and the duration for which the drug was taken. Although these cases are less common than the characteristic ON etiologies, these causes are important to know as potential causes when the clinical picture portrays no other plausible cause of ON symptoms.

Comparison of clinical studies of treatments for ON

Treatment of ON generally follows three progressive stepwise modalities of treatment: intravenous glucocorticoids acutely followed by a maintenance dosing of oral glucocorticoids, plasmapheresis, and intravenous immunoglobulin [22]. These treatment modalities may sometimes be combined. Specific modalities can be indicated more due to a patient's demographic profile. These treatment options are discussed in this review distinguishing between mechanism of action, indications, and contraindications. Intravenous glucocorticoids followed by a course of oral glucocorticoids have been the first line of acute NMOSDs before empirical evidence of efficacy related to the anecdotal experience of physician's treatment of patients [5].

A pivotal study of the effects of this treatment on patients was conducted in 1992 by the Optic Neuritis Study Group and showed, using a multicenter, randomized clinical trial, intravenous methylprednisolone followed by a course of oral prednisone restored visual acuity greatly versus a placebo group (P = .0001) [5]. It was also noted within the study that receiving just the oral prednisone versus placebo did not result in any significant (P > .05) increases in any measured outcome in the study. Patients studied were between ages 18 and 46 and mostly consisted of white females. Common side effects of the control group were sleeping disturbance, mild mood change, stomach disturbances, facial flushing, and weight gain due to the use of glucocorticoids. The goals of acute treatment are to minimize damage to the neurological structures and improve visual function. Relapses in the condition should be managed by a 1 gram daily infusion of intravenous glucocorticoids for 3-7 days, followed by an oral dosage of glucocorticoids to taper [63]. Plasmapheresis has been used for the treatment of patients with NMOSDs as well as other types of demyelinating neurological orders, either as a first line of treatment in conjunction with administered glucocorticoids or as a separate treatment for patients not responding to treatment with intravenous glucocorticoids followed by a glucocorticoid. However, there have been no large-scale randomized clinical trials to date determining the efficacy of this, specifically for NMOSDs [22,64].

A study in 2001 from Brian Weinshenker, Mayo Clinic, Department of Neurology, created a patient group who had failed to respond to treatment of their inflammatory demyelinating disease treated with a traditional regime of glucocorticoids and treated them with a small-volume plasma exchange alternating days for two weeks versus a sham trial. Masked physicians noted a moderate to marked increase in improvement of condition versus the sham group [64]. It has been determined that plasmapheresis should be used in conjunction with the administration of glucocorticoids as an adjunctive therapy or as a treatment for patients who fail to respond to glucocorticoid treatment regimes. Intravenous immunoglobulin G (IVIgG) is another treatment modality for NMOSDs. The rationale behind the treatment with IVIgG is to treat NMO associated with a marked increase in AQP4 by potentially suppressing B cell function and because of its similarity to other autoimmune diseases of this nature [65]. However, in theory, IVIgG presents as a promising modality of treatment for patients with resistant ON that fail to respond to glucocorticoids and plasmapheresis treatment [66]. Further studies are needed to further explore the effects of IVIgG, its implication as a primary modality of treatment, and as a mainstay treatment for resistant ON (Table 2).

**Table 2:** Treatment Comparisons Between Typical and Atypical Optic Neuritis ON: Optic neuritis; IV: intravenous; IVIgG: intravenous immunoglobulin G; NMOSD: neuromyelitis optica spectrum disorder; AQP4: aquaporin-4

Author (Year)	Optic Neuritis Type	Treatment Method	Groups Studied	Intervention	Results and Findings	Conclusions
Beck et al. (1992) [5]	Typical	Glucocorticoids	Multicentered, randomized clinical trial. Patients were between the ages of 18-46 and have had a history of acute unilateral optic neuritis.	Groups were treated with IV methylprednisolone 250 mg every six hours for three days, followed by oral prednisone 1 mg /kg for 14 days.	Return of vision to normal was higher that in the IV methylprednisolone group than for the placebo (P = .0001 visual field; P = .02 for contrast sensitivity; and P = .09 for visual acuity).	IV methylprednisolone is a promising treatment for restoring visual acuity in ON.
Weinshenker (2001) [65]	Typical	Apheresis	Parallel Designed Controlled Clinical Trial with 22 patients with acute attacks of MS. Patients between 18 and 60 years.	Assigned either sham or true apheresis treatment (54 ml/kg or 1.1 plasma volumes).	Favorable for apheresis. Side effects included anemia. Nine patients saw a marked increase in condition (8/19 experimental group, 1/17 sham group).	
Kleiter et al. (2018) [67]	Atypical	Apheresis	Retrospective cohort study based on the registry of German Neuromyelitis Optica Study Group, 186 patients with 1,124 attacks.	Apheresis was used as a first-line treatment in 72 patients and second line in 101 patients.	Complete remission was observed when used as a first-line therapy (OR 12.271; 95% CI: 1.04-144.91; p = .047).	Apheresis for NMOSD attacks can be beneficial, especially if the recipients are AQP4 positive.
Altunrende et al. (2019) [68]	Atypical	IVIgG	Retrospective review of 86 NMSOD patients.	Patients were treated with an initial dose of IVIgG of .4 g/kg/day.	Out of nine patients, eight had no change in visual acuity. One patient's visual acuity improved from .1 to .7.	IVIgG may be a good option after IV glucocorticoids and apheresis have failed.
Gal et al. (2015) [69]	Typical	Corticosteroid	Corticosteroid efficacy evaluated using meta-analysis of several databases.	Six clinical trials that were assessed with a total of 750 participants.	Among the meta-analyses, there is not a conclusion of whether or not glucocorticoids should be used for recovery of visual acuity induced by optic neuritis.	

## Conclusions

ON is caused by several underlying disease processes affecting clinical presentation, diagnosis, treatment, and prognosis. Acute monocular vision loss with painful extraocular movement is indicative of typical ON, whereas binocular vision loss is more commonly associated with the atypical forms. Similarly, optic disc edema is often mild in typical ON and MOGAD but severe in NMOSD. Recent advances in serological tests and diagnostics have assisted physicians with further diagnosis and treatment of this inflammatory disease to minimize nerve damage and maximize vision preservation. Due to advances in facilitating treatment, overall visual prognostics for MS or MOGAD-associated ON are generally positive, while NMOSD-related ON has demonstrated worse visual recovery. In typical ON, MRI illustrations are focally located within the orbital and canicular space, as opposed to atypical ON, where the lesion is longitudinally located and most commonly intraorbital. Further specifics of the MRI findings, such as perineuritis or orbital fat involvement are common in the atypical cause MOGAD-associated ON but are rarely seen in both typical ON and NMOSD-associated ON. Upon a positive diagnostic test, it is imperative to treat emergently with the following three modalities: intravenous glucocorticoids, plasmapheresis, and IVIgG, with intravenous glucocorticoids being an optimal primary choice. While this review is comprehensive, more studies are needed in the area of IVIgG efficacy as a primary treatment.
